# Whole-genome sequence of *Metarhizium anisopliae* JEF-157 against Asian tiger mosquito larvae (*Aedes albopictus*)

**DOI:** 10.1128/mra.00930-24

**Published:** 2024-11-27

**Authors:** Heo Ri Kim, Jae Su Kim, Se Jin Lee

**Affiliations:** 1 Department of Agricultural Life Science, Sunchon National University, Suncheon, South Korea; 2 Department of Agricultural Biology, Jeonbuk National University, Jeonju, South Korea; 3 Department of Agricultural Convergence Technology, Jeonbuk National University, Jeonju, South Korea; University of California Riverside, Riverside, California, USA

**Keywords:** *Metarhizium anisopliae*, whole-genome sequencing

## Abstract

*Metarhizium anisopliae* JEF-157, one of the biological control agents, was obtained from the Insect Microbial Biotechnology Laboratory at Jeonbuk National University. Herein, we report the whole genome of JEF-157, which has pathogenicity against Asian tiger mosquito larvae. The genome has 13,725 genes with a length of 17,719,696 bp.

## ANNOUNCEMENT

*Metarhizium anisopliae* is a microbial agent capable of controlling various pests, such as mosquitoes and locusts, and is one of the representative entomopathogenic fungi that causes diseases in insects (1–3). This strain is distributed worldwide and is primarily isolated from insect carcasses and soil (4, 5). Here, we provide the whole-genome sequencing data of *M. anisopliae* JEF-157, which exhibits high insecticidal activity against *Aedes albopictus* larvae.

*M. anisopliae* JEF-157 was obtained from the Insect Microbial Biotechnology Laboratory at Jeonbuk National University and was isolated from soil collected in Korea (Ulleung, 37°30′15.9″N, 130°51′21.2″E). This strain has been shown in previous studies to have high virulence against the fall armyworm (*Spodoptera frugiperda*)(6). The strain was cultured on SDA/4 (Sabouraud Dextrose Agar) at 25°C ± 1°C for 14 days for whole-genome sequencing analysis. The genomic DNA of *M. anisopliae* JEF-157 was extracted by the Wizard Genomic DNA Purification Kit (Promega, USA). Genome sequencing of *M. anisopliae* JEF-157 was conducted using the Illumina NovaSeq 6000 (Illumina, USA) and PacBio Sequel IIe (Pacific Biosciences, CA, USA) platforms by Macrogen (Seoul, South Korea).

The DNA libraries were generated with the TruSeq Nano DNA High Throughput Library Prep Kit (Illumina, USA) and sequenced. The library preparation utilized the PacBio SMRTbell prep kit 3.0. For annealing the SMRTbell templates, the Int Ctrl 3.2 and Sequel II Bind Kit 3.2 were used. Sequencing was then carried out using the Sequel II Sequencing Kit 2.0 and the SMRT Cell 8M Tray. The reads generated by the PacBio Sequel IIe system were assembled using SMRT Link 11.1.0.166339. Assembly quality was improved using Pilon version 1.21 with high-quality adapter-trimmed Illumina reads (7).

The PacBio Sequel IIe sequencing generated 2,153,210 HiFi reads (31,547,731,004 bases; *N*_50_, 15,280 bp), and filtered Illumina NovaSeq sequencing generated 36,473,748 reads (5,504,996,281 bases; G + C content, 49.89%). As a result of mapping the reads to the assembled contigs and performing polishing with Racon, a higher-quality consensus sequence was generated. The assembly output consists of primary contigs and haplotigs. Primary contigs represent the longest continuous sequences assembled, while haplotigs capture alternative haplotype sequences, which are commonly observed in polyploid genomes. The assembly was polished three times with the Illumina reads. Following this, coverage depth was estimated by aligning the HiFi reads to the assembled contigs. During this process, contigs shorter than 1 kb (in both primary contigs and haplotigs) were removed. The final assembly contained 179 contigs with a total length of 47,261,936 bp and an average depth of 620.5×. Additionally, 103 haplotigs with a total length of 2,265,597 bp and an average depth of 177.0× were obtained.

For gene prediction, a gene training model was created using SNAP (28 July 2006), and gene prediction was performed using Maker 3.01.03 (8). Genome completeness was evaluated using BUSCO version 5.1.3 (9), and the analysis was performed with eukaryota_odb10. For single-copy complete BUSCO(S), a score of 84.71% was recorded, while for duplicated complete BUSCO(D), the score was 14.12% (missing BUSCO[M], 0%; total BUSCO groups searched, 255). Additionally, for further annotation, InterProScan version 5.30-69.0 (10) and psiblast version 2.4.0 (11) were performed using the EggNOG DB version 4.5 (12). As a result of the annotation, a total of 13,725 genes were identified with a genome length of 17,719,696 bp and a protein length of 5,849,672aa. This study has obtained genomic information, including insecticidal genes such as chitinase, destruxin, protease, lipase, and lectin from *M. anisopliae* isolated from Korean soil. These data will contribute to understanding the insecticidal mechanisms against mosquitoes.

## Data Availability

The complete genome sequence of *Metarhizium anisopliae* JEF-157 (GenBank accession number JBFPET000000000) has been deposited under BioProject accession number PRJNA1136439. The raw reads are available in the Sequence Read Archive (SRA) accession numbers SRR29856235 and SRR29856236.
